# Medical History from the Earliest Times

**Published:** 1892-11-05

**Authors:** E. T. Withington


### MEDICAL HISTORY FROM THE EARLIEST TIMES.

By E. T. Withington, M.A., M.B. Oxon.

### XXX.—ARABIC MEDICINE. (3) HALY ABBAS AND AVICENNA—*(Continued).*

Among the works ascribed to Isaac ben Solomon is one called "Pantegni," or "Pantechnum," which is practically identical with the famous "Maleki" or "Royal Book" of Haly Abbas. This resemblance can only be explained by Arabic scholars, and their authority is unfortunately divided between two hypotheses, (1) that Haly Abbas simply copied Isaac's book, substituting his own name for that of the author, and (2) that the work of the Moslem physician has been attributed by some strange mistake to his Jewish predecessor. It is hard to believe that a great work by so famous a writer as Isaac Judæus could have been republished as original a generation later without the fraud being at once discovered, and the second and more usual explanation will be here adopted.

Ali ben al-Abbas (†994), whose Persian origin is indicated by his title al-Madjoussy, "the Magus," is the second of the great Moslem physicians, and he intended his "Royal Book" to form a complete cyclopædia of medicine, all previous works of that nature being more or less imperfect, as he points out at length in the introduction. The "Maleki" comprises twenty books, ten theoretic, and ten practical, and though founded on Galen, is by no means destitute of originality. Haly gives due weight to personal experience, and urges the student to be diligent in attending hospitals, and the practice of good physicians. In contrast to Michael Mesue, who never employed any drug which had been in use for less than two centuries, Haly Abbas insists on the importance of searching for new remedies, but he points out at the same time the necessity for caution in such experiments, and advises, among other things, that they should be first given to animals. As a specimen of the "Maleki," we may take the 25th chapter of the fifth practical book "On the Treatment of Love," which Haly considers a form of melancholia. "Such patients should undergo a moistening regimen (black bile as we have seen corresponded to the quality 'dryness'). They should take baths, moderate horse exercise, and anoint themselves with oil of violets. They should look upon gardens, fields, meadows, and flowers, listen to sweet and low sounds as of the lute or lyre, and their minds should be occupied by stories, or pleasant and interesting news. But they must also have some work or business, so as to keep them from idleness, and from thoughts of the loved ones; and it is good to excite them to quarrels and arguments, that their minds may be yet further distracted. Let them also cultivate the acquaintance of other young women." In the theoretical part Haly Abbas observes that both sexes are prone to melancholia at the period of adolescence. The "Royal Book" was translated into Latin in 1080 by Constantine, the African, who published it as his own composition under the name Pantegni, and in 1127 by Stephen of Antioch, from whose version the above extract is taken.

Haly Abbas, if not himself a plagiarist, is the most unfortunate of medical authors, for he has not only suffered the strange fate of being robbed by a predecessor, as well as by a successor, but he was immediately followed by another writer, who, though scarcely greater as a physician, far surpassed him in the renown of universal learning, and the "Maleki" was rapidly superseded, both among Arabs and Christians, by the "Canon" of Avicenna.

Abu Ali al-Husain ben Abdallah eben Sina is the foremost representative of Moslem science, and is rarely named by his countrymen without the honourable epithets al-Scheik, al-Reis, the Revered, the Prince. He has himself told us the story of his early life. Born in the province of Bokhara, A.D. 980, he could repeat the Koran by heart when ten years old. The art of healing seemed to him a comparatively easy study. "Medicine is no hard and thorny science, like mathematics and metaphysics, so I soon made great progress; I became an excellent doctor, and began to treat patients, using approved remedies. . . . At twelve years of age I disputed in law and logic." From twelve to sixteen he studied day and night, sometimes falling into a slumber and solving problems in his dreams. "When I found a difficulty, I referred to my notes and prayed to the Creator. At night, when weak or sleepy, I strengthened myself with a glass of wine." This latter habit unfortunately grew upon him, and Barhebraeus somewhat rashly asserts that

Avicenna was the first philosopher addicted to strong drink. At sixteen he had mastered everything except the "Metaphysics" of Aristotle. Forty times did he read that abstruse work till he knew the words by heart, but was no nearer to their meaning. But one day being offered the commentary of Abu Nasr al Farab at the ridiculously cheap price of three dirhems (eighteenpence), he bought it, and found there the key of the mystery. Then, having given thanks to God and alms to the poor, he started on his travels. Passing over forty years of an adventurous life, we find the great physician, worn out by excesses physical and mental, dying at Hamadan. When he saw that medicines had no more effect, he resigned himself to the inevitable, sold his goods and gave to the poor, and, betaking himself to his bed, read the Koran once through every three days, till he died in the holy month Ramadan—June, 1037.

Avicenna was not only a physician, but also an astronomer, poet, philosopher, and statesman. Of the 100 works ascribed to him only about twelve are medical, but as few besides these have been translated into European languages, it is as a physician that he is usually estimated. His chief medical book, the "Canon," not only superseded the similar works of Rhazes and Haly Abbas, but, when translated into Latin, overshadowed for a time even the writings of Galen, and for four centuries formed the chief text-book of European medicine. Yet the "Canon" is admitted even by the Arabs themselves to be inferior in practical value and originality to the "Maleki" and the "Continens," and its Latin translation is so dry and tedious that even the indefatigable Haller, who read the "Continens" with pleasure, admits his inability to get through the "Canon," which he characterises as a "methodica inanitas." The reader may be content with the shortest and one of the most generally useful of its innumerable chapters. Book iii., Fen. vi., tract iii., cap. xx., "On driving away moths: Wormwood protects cloths from moths, likewise calamint, likewise lemon-peel." But let us not, therefore, despise the work, still less its author; but rather look for the reasons for its wonderful success. Avicenna was greater as a philosopher than as a physician, and his object in the "Canon" was to reconcile the doctrines of Aristotle with those of Galen, just as St. Thomas Aquinas, two centuries later, reconciled them with those of the Catholic Church. Thus, in speaking of the causation of diseases, he discusses not only the primitive (exciting), antecedent (predisposing), and conjoint (proximate) causes of Galen, but also the material, formal, effectual, and final causes of Aristotle, a combination which could not fail to impress the mediæval physician, who held that where Galen and Aristotle differed none could decide, and that where they agreed none could dissent.

Avicenna's scientific works are, perhaps, more worthy of his fame, and the treatise "On the Uselessness of Astrology," had it been translated, might have benefited medicine more than the "Canon." If the work "On Petrifactions" is really by him, Avicenna may claim the title of Father of Geology, and the following extract might have been published as something new more than eight centuries later: "On the Origin of Mountains.—Mountains are produced in two ways, either by elevations of the earth's crust, as in earthquakes, or by the action of water, which has hollowed out the valleys at the same time; for there are harder and softer tracts, and wind and water remove the latter while leaving the former. Many ages (*multa tempora*) have been required to produce this, and perhaps the mountains are now getting smaller. That water has been the chief agent is shown by the marks of aquatic and other animals found on many rocks."

